# Adjustment Disorder in Older Adults: Insights from a Nationwide Survey

**DOI:** 10.1093/geroni/igaf122.3043

**Published:** 2025-12-31

**Authors:** Sang Hui Chu, Subin Park, Hun Kang, Ocksim Kim

**Affiliations:** Yonsei University, Seoul, Republic of Korea; Yonsei University College of Nursing, Seoul, Republic of Korea; Yonsei University, Seoul, Republic of Korea; Yonsei University College of Nursing, Seoul, Republic of Korea

## Abstract

Adjustment disorder (AjD) is a stress-related condition that varies across age groups. Younger adults frequently develop AjD due to work and educational stressors, whereas older adults face challenges such as declining health and social isolation. This study compared the prevalence and risk factors of AjD in adults aged 60 and above versus those aged 59 and younger using the International Adjustment Disorder Questionnaire (IADQ). A total of 916 participants from a nationwide online survey were analyzed, with older adults (>60 years, n = 255) and younger adults (< 60 years, n = 661). The overall prevalence of AjD was 20.5%, with a significantly lower rate in older adults (11.4%) compared to younger adults (24.1%, p<.001). Younger adults were more likely to report work-related (53.3% vs. 28.2%, p<.001) and educational stressors (23.9% vs. 7.1%, p<.001), whereas older adults were significantly more affected by health-related stressors (67.8% vs. 51.3%, p<.001). Logistic regression showed that an increasing number of stressors significantly predicted AjD in younger adults (e.g., OR = 3.900 for five stressors, p<.001). In contrast, residing in the capital region was a significant risk factor for older adults (OR = 4.130, p=.004). Anxiety symptoms were the strongest common predictor of AjD across both groups, with a greater effect in older adults (OR = 21.310, p=.030). These findings highlight the unique vulnerabilities of older adults in developing AjD, particularly to health-related stressors and urban living. Age-specific interventions should prioritize mental health support tailored to older adults, with a focus on mitigating health concerns and enhancing social well-being.

